# The Diseases of the Ear and Their Treatment

**Published:** 1887-03

**Authors:** 


*The Diseases of the Ear and their Treatment.* By ARTHUR HARTMANN, M.D., Berlin. Translated from the Third German Edition by JAMES ERSKINE, M.A., M.B. pp. 283. Edinburgh: YOUNG J. PENTLAND. 1887.

Dr. Erskine has earned the thanks of all English students by translating this concise and practical work, which in its original language has rapidly reached a third edition.

The book is prefaced by a very interesting historical sketch of the treatment of ear diseases, commencing with "an ancient papyrus scroll preserved in the Egyptian collection of the Berlin Museum, and supposed to date from the reign of Rameses II., who adopted the Jewish lawgiver, Moses, *i.e.* the fourteenth century B.C." The author's arrangement of subjects is methodical and clear. Commencing with a chapter headed "Diagnostic," he passes on through "Symptomatology," "Frequency, Ætiology, and Prophylaxis of Ear Diseases," and "General Therapeutics," to the chapters on particular ear diseases; while the translator has added an appendix, containing a list of instruments and the therapeutical formulæ in a form for easy reference.

Chapter iii. is a particularly good one, and is full of practical suggestions for the practitioner. Thus, on p. 64 we read: "Under ordinary conditions, the healthy ear does not need to be protected from cold; only during extreme cold or stormy and rainy weather ought cotton-wool to be inserted, into children's ears especially. The same precaution must be taken in the case of every ear predisposed to inflammation. All persons whose membranæ are perforated ought to protect their ears with cotton-wool. The entrance of cold fluids into any ear must always be prevented; and so, while bathing or diving, the ear ought to be plugged. Patients with perforations of the membrane should be very careful in this respect, as violent inflammation may be caused by the entrance of cold water."

In speaking of the removal of plugs of cerumen, the author mentions that tepid water has been found a better solvent for this secretion than any other fluid, such as oil, brandy, or lime-water; but he speaks with approval of the addition of a little soda to the water.

He classes inflammations of the middle ear into acute and chronic, further subdividing the former into acute catarrh and acute purulent inflammation, and the latter into chronic catarrh and chronic purulent inflammation; this arrangement strikes us as simpler and better for all practical purposes than any other which has yet been suggested.

He divides polypi into three kinds; namely, *granulations*, *fibromata*, and *myxomata;* and says that granulations are most frequently observed, forming 55 per cent. He recommends the wire snare for their removal, and mentions chromic acid as the best caustic for applying to the stumps.

He approves of the antiseptic treatment of diseases of the middle ear with suppuration; but dismisses one popular remedy with the curt remark (on p. 184) that "iodoform has proved bad."

We are surprised to observe that, in a work which is otherwise so well up to date, so little allusion should be made to trephining for intra-cranial suppuration, the result of ear disease. The author simply says that "Schede brought about a cure in the case of an abscess in the temporal bone by opening it from the outside." We fancy this must have been written some years ago.

He does not look upon Menière's disease as due to a definite lesion, but speaks of it as "Menière's complex of symptoms," and says it may be due to—

1. Stimuli from the middle ear.
2. Disease of the labyrinth.
3. Pathological processes in the brain.

The work of the translator has been performed with great ability, upon the whole, although there are a few blemishes. Thus, on p. 142 we read: "When the renewal of air between the tympanic cavity and the atmosphere through the tubes ceases, the quantity of air contained in the tympanum diminishes due to exchange of gases, and resulting in a preponderance of atmospheric pressure upon the outer surface of the membrana tympani, driving it inwards."

The name of the publisher is a sufficient guarantee for i part of the work.

